# A Substance P (SP)/Neurokinin-1 Receptor Axis Promotes Perineural Invasion of Pancreatic Cancer and Is Affected by lncRNA LOC389641

**DOI:** 10.1155/2022/5582811

**Published:** 2022-05-12

**Authors:** Tengfei Ji, Keqiang Ma, Hongsheng Wu, Tiansheng Cao

**Affiliations:** Department of Hepatobiliary Surgery, Affiliated Huadu Hospital, Southern Medical University (People's Hospital of Huadu District), Guangzhou, China

## Abstract

Perineural invasion (PNI) is considered to be a main reason for the poor prognosis of pancreatic cancer. In the present study, we analyzed the roles of substance P (SP)/neurokinin-1 receptor (NK-1R) and lncRNA LOC389641 in pancreatic cancer PNI. Pancreatic cancer cell lines BxPC-3 and MIAPaCa-2 were cocultured with SH-SY5Y cells and then stimulated with SP to simulate the *in vivo* influence of ganglia on pancreatic cancer. The BxPC-3 and MIAPaCa-2 cells were transfected with a neurokinin-1 receptor (NK-1R) overexpression vector, NK-1R silencing vector, LOC389641 overexpression vector, or LOC389641 silencing vector, respectively. The proliferative abilities of BxPC-3 and MIAPaCa-2 cells were assessed using the cell counting kit-8 and 5-ethynyl-2′-deoxyuridine (EdU) assays. Wound-healing and Transwell assays were performed to determine the migration and invasion abilities of the cells. When SP was added to the coculture system, it positively regulated cancer cell proliferation, migration, and PNI and significantly activated the NK-1R/Akt/NF-*κ*B signaling pathway. Incubation with 100 nmol/L SP for 24 h was selected as the optimal condition for treatment. The activated NK-1R positively regulated the proliferation, migration, and invasion of pancreatic cancer cells. However, the levels of lncRNA LOC389641 and tumor necrosis factor receptor SF10A (TNFRSF10A) mRNA in BxPC-3 and MIAPaCa-2 cells were not affected by SP treatment. Overexpression or silencing of LOC389641 changed the effect of SP stimulation on pancreatic cancer PNI. When taken together, these results revealed that SP/NK-1R and LOC389641 promoted the progression of pancreatic cancer PNI. Moreover, we found that pancreatic cancer PNI promoted by the SP/NK-1R axis could be blocked by the TNFRSF10A/NF-*κ*B pathway mediated by LOC389641.

## 1. Introduction

Pancreatic cancer is one of the deadliest malignant tumors and accounted for 432,242 cancer-related deaths in 2018 [1]. Moreover, the incidence of pancreatic cancer continues to increase on a yearly basis [2]. Pancreatic cancer is characterized by a scarcity of early symptoms, resistance to treatment, a poor prognosis, and a high mortality rate [2–4]. Moreover, the chronic symptoms and severe abdominal pain caused by pancreatic cancer significantly affect a patient's quality of life [5]. While the methods for treating many cancers have substantially improved in recent decades, the treatment outcomes of patients with pancreatic cancer have not significantly improved.

Perineural invasion (PNI) is a prominent characteristic of pancreatic cancer and occurs in almost all pancreatic cancer patients [6, 7]. PNI refers to the process by which cancer cells invade surrounding nerves and is observed during the early stage of pancreatic cancer. PNI is associated with pain, a high rate of tumor recurrence, and a lower overall survival rate [8]. PNI is defined as a characteristic of malignant tumor behavior and is associated with a poor prognosis for pancreatic cancer patients.

With the development of new molecular treatments, PNI has been widely studied in terms of mRNA, long noncoding RNAs (lncRNAs), and signalling pathways. Studies have demonstrated that PNI can be reduced by targeting signaling pathways, and those findings have suggested new strategies for treating pancreatic cancer [9, 10].

Neurokinin-1 receptor (NK-1R) is a tachykinin receptor distributed in peripheral tissues and the nervous system; it is involved in various immune responses, neurogenic inflammation, pain, and depression [11]. After combining with substance P (SP), the SP/NK-1R participates in cancer pathophysiological processes [12–14], by regulating tumor cell proliferation, migration, metastasis, and angiogenesis [15]. The SP/NK-1R axis acts by modulating various signaling pathways, including the extracellular-regulated MAP kinase (ERK), wingless-type MMTV integration site family (Wnt), and serine/threonine kinase (Akt) pathways. Therefore, it is important to study the roles played by SP/NK-1R in regulating the signaling pathways involved in pancreatic cancer. lncRNAs have been widely studied in recent years. LOC389641 was reported to promote pancreatic ductal adenocarcinoma progression and increase cell invasion by regulating E-cadherin, with the possible involvement of tumor necrosis factor receptor SF10A (TNFRSF10A) [16]. However, it remains unknown whether LOC389641 affects pancreatic cancer via the SP/NK-1R axis. We found that SP/NK-1R signaling and LOC389641/TNFRSF10A may share the same target, i.e., nuclear factor of kappa light polypeptide gene enhancer in B cells, p65 (NF-*κ*B p65). The present study sought to identify factors that influence pancreatic cancer progression via SP/NK-1R and the LOC389641, as well as its target gene, *TNFRSF10A*. Our results identified a possible target for treating pancreatic cancer.

## 2. Materials and Methods

### 2.1. Cell Culture

Pancreatic cancer cell lines BxPC-3 and MIAPaCa-2 were purchased from ATCC (Manassas, VA, USA) and cultured in RPMI 1640 medium supplemented with 10% fetal bovine serum at 37°C in a 5% CO_2_ atmosphere. Human SH-SY5Y neuroblastoma cells were acquired from the Kunming Institute of Zoology (Kunming, China) and cultured in the same medium as described above. SP was obtained from MCE (Shanghai, China). *In vitro* analyses were performed on BxPC-3 (BxPC-3Ctrl) and MIAPaCa-2 (MIAPaCa-2Ctrl) cells and cells cultured with SH-SY5Y (SH-SY5Y-Vector), as well as cells cultured with SP and SH-SY5Y (SH-SY5Y-SP). For coculture, BxPC-3 or MIAPaCa-2 cells were seeded into the lower chamber of a Transwell plate, while the upper chamber was seeded with SH-SY5Y cells.

For selection of the optimal SP concentration, BxPC-3 and MIAPaCa-2 cells were incubated with SP at concentrations of 0, 10, 25, 50, 100, and 150 nmol/L for 24 h. After selecting the concentration of 20 nmol/L, the optimal incubation time was selected by performing incubations for 0 h, 12 h, 24 h, 36 h, and 48 h, respectively.

### 2.2. Transfections

BxPC-3 and MIAPaCa-2 pancreatic cancer cells were treated with 100 nmol/L SP for 24 h and then transfected with an NK-1R overexpression vector (NK-1R + SP group), NK-1R silencing vector (siNK-1R + SP group), LOC389641 overexpression vector (LOC389641 + SP group), or LOC389641 silencing vector (si-LOC389641 + SP group), respectively. SiRNA targeting NK-1R (5′-UUU UCC UUC UCU UUU GCU GCC-3′) and siRNA targeting LOC389641 (5′-AGGAAAAUCCAGAAGAAAGGC-3′) were synthetized by Ribobio (Guangzhou, China).

The pGL3-Basic vector (Promega Corporation, Madison, WI, USA) was used for transfections. The NK-1R DNA promoter sequences (forward primer: 5′-GGGGTACCTCTACCGTTTGAAATGGTCTTG-3′ and reverse primer: 5′-CCCTCGAGGCAGTAGGTGGCTTGGAAGTA-3′) and LOC389641 DNA promoter sequences (forward primer: 5′-GGGGTACCTGGGAGGTGGGTAGTAGGG-3′ and reverse primer: 5′-CCCTCGAGTGACACGGGATGAGACTTGG-3′) were designed by Sangon Biotech (Shanghai, China). All transfections were carried out by using Lipofectamine 2000 (Invitrogen, Waltham, MA, USA) according to the manufacturer's instructions.

### 2.3. Enzyme-Linked Immunosorbent Assay (ELISA) Assay

Cell supernatants were collected after treatment with reagents. SP concentrations in the Ctrl, SH-SY5Y-Vector, and SH-SY5Y-SP groups were measured by using standardized ELISA assays (Boster, Wuhan, China) according to the manufacturer's instructions.

### 2.4. Cell Proliferation Analysis

The effect of SP on the proliferative ability of BxPC-3 and MIAPaCa-2 cells was assessed using the cell counting kit-8 (CCK-8; Glpbio, Montclair, CA, USA) and 5-ethynyl-2′-deoxyuridine (EdU; EdU Staining Proliferation Kit; Abcam, England) assays. The cells were cultured with CCK-8 reagent for 2 h at 37°C, and the absorbance of each culture well at OD_450nm_ was determined at 0 h, 12 h, 24 h, 36 h, and 48 h using a microplate reader (Biotech, China).

An EdU Proliferation Assay Kit (iFluor 647) (#ab222421; Abcam, Cambridge, MA, UK) was used to detect the proliferation rates of BxPC-3 and MIAPaCa-2 cells. In brief, 5 × 10^5^ cells were cultured for 2 h in 15 mL tubes that contained culture medium supplemented with 20 m*Μ* EdU reagent. After culture, the cells were fixed with paraformaldehyde and stained with 4′,6-diamidino-2-phenylindole (DAPI). Finally, the percentage of EdU-positive cells was analyzed and quantified by time-lapse fluorescence microscopy (Lonheart FX Automated Microscope, Winooski, VT, USA).

### 2.5. Wound-Healing Analysis

Approximately 3 × 10^5^ BxPC-3 and MIAPaCa-2 cells were seeded into a 6-well plate and cultured to ~80% confluence. Next, a straight line was scratched in each well using a 10 *μ*L pipette tip. One day later, a microscope equipped with a camera was used to record the cell migration distance, and the migration rate was analyzed.

### 2.6. Transwell Analysis

Cell migration and invasion analyses were performed using 24-well Transwell plates according to the manufacturer's instructions. Briefly, approximately 1 × 10^4^ BxPC-3 and MIAPaCa-2 cells were added to the upper chamber of a Transwell plate (Corning, NY, USA) that was either precoated or not coated with Matrigel matrix (BD, Franklin Lakes, NJ, USA). After 24 h, the supernatants of cultured SH-SY5Y cells were collected and added to the Transwell chambers. After culture for 24 h, the culture medium was replaced with basal medium without FBS, and the Transwell plate was incubated for 4 h. Finally, culture medium containing 10% FBS was added to the lower chamber. After further culture, the invaded cells were fixed, stained, observed, and counted under an Olympus microscope at 40x magnification.

### 2.7. Quantitative Real-Time PCR (qRT-PCR) Analysis

The levels of *NK-1R* gene expression in the SH-SY5Y-SP group treated with SP at concentrations of 0, 10, 25, 50, 100, and 150 nmol/L were assessed after 0 h, 12 h, 24 h, 36 h, and 48 h of treatment. Moreover, the levels of NK-1R, LOC389641, and TNFRSF10A expression were assessed in pancreatic cancer cells and transfected cells.

The total cellular RNA was extracted using TRIzol Reagent (Invitrogen, USA). The primers for NK-1R, LOC389641, and TNFRSF10A were designed using Primer5software and synthesized by Sangon Biotech (Shanghai, China) (Table S1). A HiScript II One Step qRT-PCR SYBR Green Kit (#Q221-01; Vazyme Co., Ltd.; Nanjing, China) was used to evaluate the relative level gene expression.

### 2.8. Western Blotting

The total proteins in cells were extracted using RIPA lysis buffer (#R0278, Sigma) and quantified using a BCA Protein Assay Kit (Beyotime, Jiangsu, China). Next, a sample of total protein from each extract was separated by standard sodium dodecyl sulfate-polyacrylamide gel electrophoresis (SDS-PAGE), and the protein bands were transferred onto PVDF membranes that were subsequently blocked with 5% nonfat milk. The membranes were then incubated primary antibodies against NK-1R (#ab25445, 1 : 00; Abcam, Cambridge, UK), NF-*κ*B p65 (#ab16502, 1 : 1000; Abcam), AKT (#ab28422, 1 : 1000; Abcam), phosphor-AKT (p-AKT) (#ab38449, 1 : 1000; Abcam), TNRSF10A (#ab157345, 1 : 1000; Abcam), GAPDH (#ab9485, 1 : 2500; Abcam), and HDAC1 (#ab41407, 1 : 1000; Abcam). Subsequently, the PVDF membranes were washed and then incubated with the corresponding HRP-conjugated secondary antibody (1 : 5000). The immunostained protein bands were observed by electrochemiluminescence (Millipore, Germany).

### 2.9. Immunofluorescence (IF) Analysis

The cells were fixed with 4% formaldehyde, blocked with 5% BSA, and then incubated with anti-NK-1R and an anti-TNFRSF10A fluorescent secondary antibody (Cell Signaling Technology, Danvers, MA, USA) for 48 h. Next, the cells were stained with DAPI and observed under a fluorescence microscope (Leica, Germany). Five randomly selected visual fields in each group were observed, photographed, and analyzed.

### 2.10. Statistical Analysis

All data were analyzed using the GraphPad Prism 8 software (GraphPad Software, La Jolla, CA, USA). One-way ANOVA or the student's *t*-test was used to make comparisons between groups. All experiments were repeated at least three times, and statistical data represent the mean value ± SD. A *P* value < 0.05 was considered to be statistically significant.

## 3. Results

### 3.1. SP Positively Regulated Cell Proliferation, Migration, and Invasion during Pancreatic Cancer PNI

**Figure 1:**
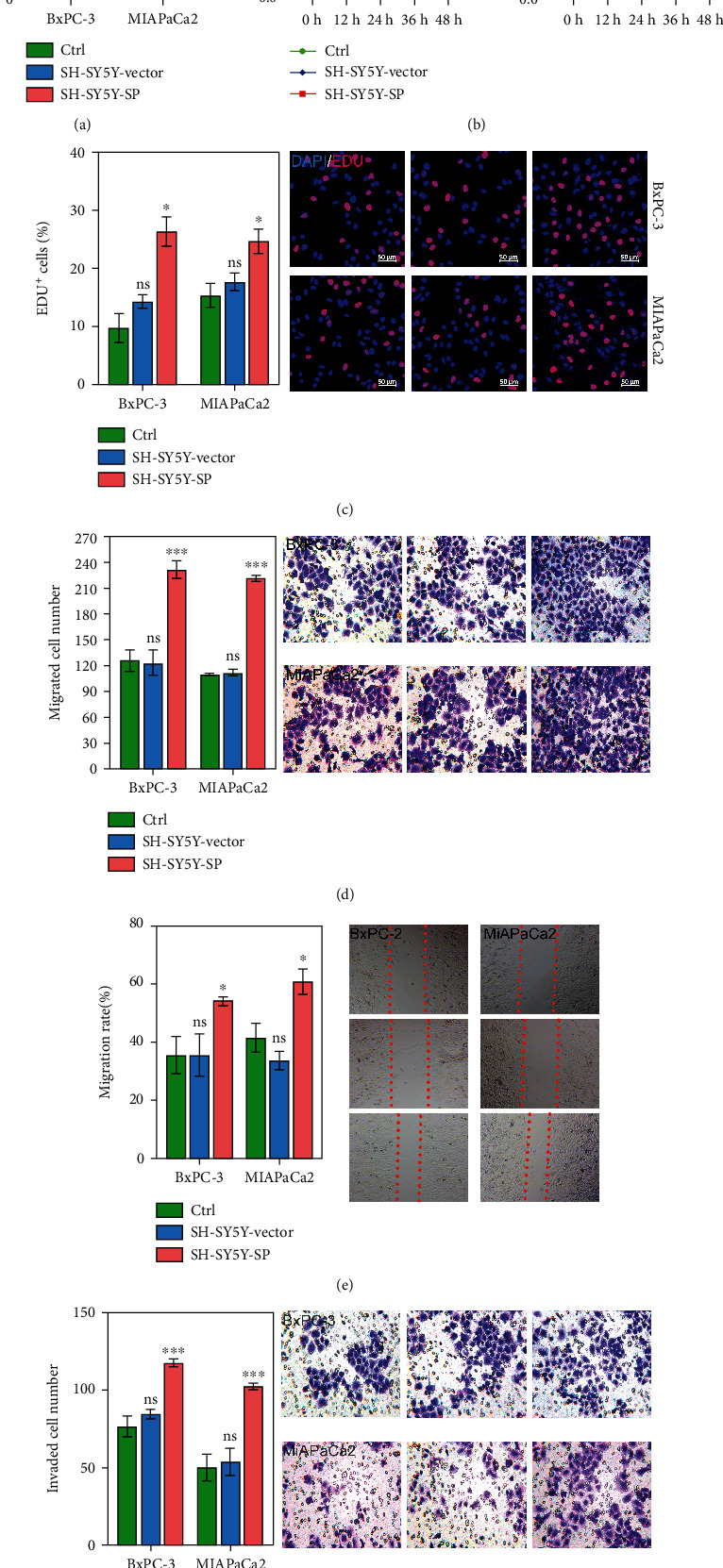
SP significantly promoted cell proliferation, migration, and invasion. (a) SP concentrations were measured using an ELISA kit. (b, c) Growth curves and proliferation rates for BxPC-3 and MIAPaCa-2 cells cultured with SP and SH-SY5Y were analyzed by CCK-8 and EdU staining assays. (d, f) Transwell assays were conducted to test the effects of SP on cell migration and invasion. (e) Wound-healing assays were performed to evaluate the cell migration rates regulated by SP. ns: no significant difference, ^∗^*P* < 0.05, ^∗∗∗^*P* < 0.001 vs. SH-SY5Y-Vector group.

To evaluate the role of SP in pancreatic cancer cells, the rates of pancreatic cancer cell proliferation, migration, and invasion were analyzed, and the effects of SP on those parameters were also examined. First, the SP concentration was evaluated in each group. As expected, the concentration of SP in the SH-SY5Y-SP group was higher than those in the Ctrl and SH-SY5Y-Vector groups (*P* < 0.05; Figure 1(a)). CCK-8 assays showed that SH-SY5Y-SP significantly increased the proliferation of BxPC-3 and MIAPaCa-2 cells as compared with proliferation in the Ctrl and SH-SY5Y-Vector groups (*P* < 0.05; Figure 1(b)). The percentages of EdU-positive cells were significantly higher in the SH-SY5Y-SP group than in the Ctrl and SH-SY5Y-Vector groups (*P* < 0.05; Figure 1(c)), indicating that SP supplementation promoted cell proliferation. The migration abilities of cells were evaluated using Transwell and wound-healing assays. When compared with cells in the Ctrl and SH-SY5Y-Vector groups, the number of migrated and invading cells in the SP groups was significantly increased (*P* < 0.001; Figures 1(d) and 1(f)). Wound-healing assays revealed that SP supplementation significantly accelerated cell migration (*P* < 0.05; Figure 1(e)). These findings indicated that SP positively regulated the proliferation, migration, and invasion of BxPC-3 and MIAPaCa-2 cells.

### 3.2. SP Significantly Stimulated the NK-1R/Akt/NF-*κ*B Signaling Pathway

**Figure 2:**
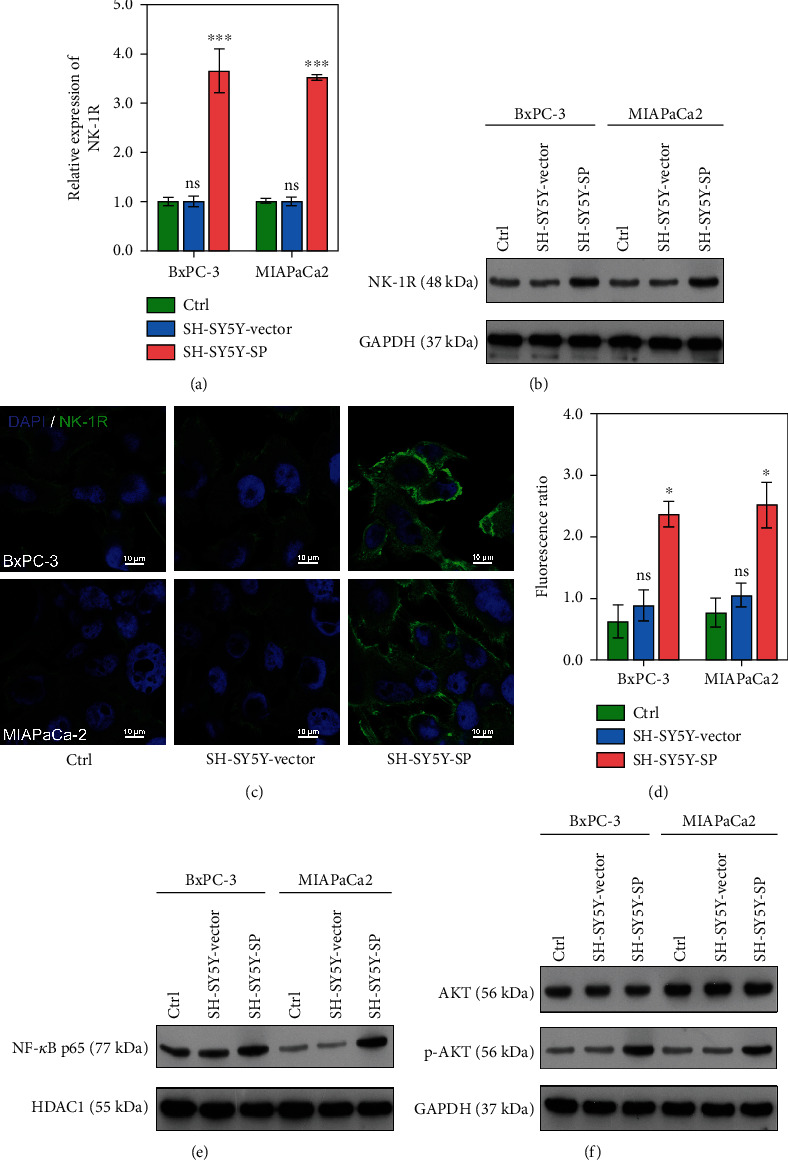
SP significantly increased the levels of NK-1R, NF-*κ*B p65, and p-AKT expression. (a–d) The levels of NK-1R expression were assessed using the qRT-PCR, western blotting, and IF. (e) The levels of NF-*κ*B p65 protein expression were evaluated using HDAC1 as a reference. (f) The levels of AKT and p-AKT proteins were evaluated using GAPDH as a reference. ns: no significant difference, ^∗^*P* < 0.05, ^∗∗∗^*P* < 0.001 vs. SH-SY5Y-Vector group.

The level of NK-1R expression was positively correlated with the concentration of SP. Therefore, we performed qRT-PCR, western blotting, and IF studies to confirm the levels of NK-1R expression in the Ctrl, SH-SY5Y-Vector, and SH-SY5Y-SP groups. Our results showed that NK-1R expression was significantly upregulated in the SH-SY5Y-SP group when compared with the Ctrl and SH-SY5Y-Vector groups (*P* < 0.001; Figure 2(a)). Furthermore, western blot studies showed that NK-1R expression was significantly increased in cells treated with SP (Figure 2(b)). As observed in Figure 2(c), the fluorescence ratio of NK-1R was significantly higher in the SH-SH5Y-SP group as compared with the Ctrl and SH-SY5Y-Vector groups (*P* < 0.05; Figures 2(c) and 2(d)). These results demonstrated that SP was positively correlated with NK-1R expression.

NF-*κ*B p65 is a transcription factor for NK-1R and might also positively regulate NK-1R. Western blot analyses were performed to determine the effect of the NK-1R/Akt/NF-*κ*B signaling pathway in cocultured cells. Therefore, we evaluated the levels of NF-*κ*B p65 expression in the Ctrl, SH-SY5Y-Vector, and SH-SY5Y-SP groups by western blotting. Our results showed that both NF-*κ*B p65 and p-AKT expression were upregulated in the SH-SY5Y-SP group as compared with the Ctrl and SH-SY5Y-Vector groups (Figures 2(e) and 2(f)). When taken together, our findings showed that SP significantly stimulates the NK-1R/Akt/NF-*κ*B signaling pathway.

### 3.3. Selection of the Optimal SP Concentration and Treatment Time

**Figure 3:**
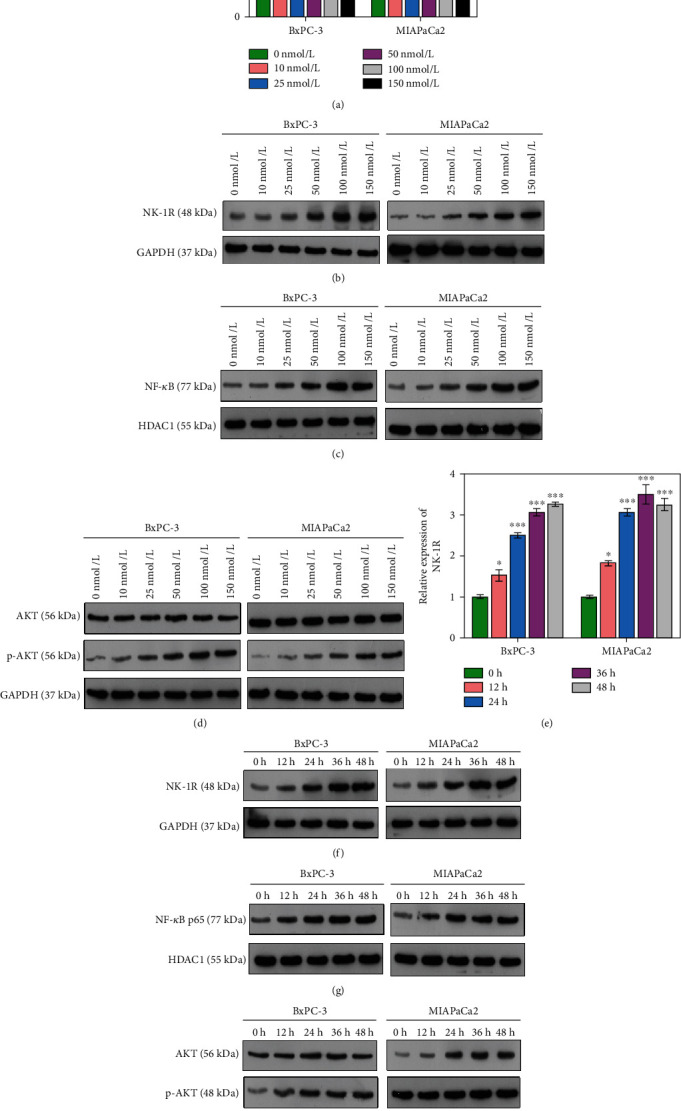
Selection of the optimal SP concentration and treatment time. (a–d) The optimal SP concentration was selected after testing concentrations of 0, 10, 25, 50, 100, and 150 nmol/L. (a) *NK-1R* gene expression at each SP concentration. (b–d) The levels of NK-1R, NF-*κ*B p65, AKT, and p-AKT protein expression at each concentration of SP. (e–h) The optimal SP treatment time was selected after incubation for 0 h, 12 h, 24 h, 36 h, and 48 h, respectively. (e) *NK-1R* gene expression at each SP treatment time. (e–h) The levels of NK-1R, NF-*κ*B p65, AKT, and p-AKT protein expression at each SP treatment time. ^∗^*P* < 0.05, ^∗∗∗^*P* < 0.001 vs. 0 nmol/L group or 0 h group.

After confirming the role played by SP in regulating NK-1R, NF-*κ*B p65, and p-AKT, the optimal SP concentration and treatment time were selected. We first examined the levels of NK-1R, NF-*κ*B p65, and AKT expression in the presence of SP at concentrations of 0, 10, 25, 50, 100, and 150 nmol/L. We found that NK-1R expression was continuously enhanced when the SP concentration exceeded 10 nmol/L. Moreover, the levels of NK-1R, NF-*κ*B p65, and p-AKT expression were significantly increased when the SP exceeded 25 nmol/L, as compared to SP concentrations of 0 nmol/L and 10 nmol/L (*P* < 0.001; Figures 3(a)–3(d)). However, the expression levels of those factors did not change when the SP concentration exceeded 100 nmol/L, indicating that 100 nmol/L was the optimal SP concentration.

We next examined how SP treatment time (0 h, 12 h, 24 h, 36 h, and 48 h) influenced the levels of NK-1R, NF-*κ*B p65, AKT, and p-AKT expression. We found that the expression of all those factors significantly increased with increasing SP treatment time (i.e., 12 h, 24 h, 36 h, and 48 hr; all *P* values < 0.05) (Figures 3(e)–3(h)). SP treatment for 24 h was selected as the optimal treatment time.

### 3.4. NK-1R Positively Regulated the Proliferation, Migration, and Invasion of Pancreatic Cancer Cells during PNI

**Figure 4:**
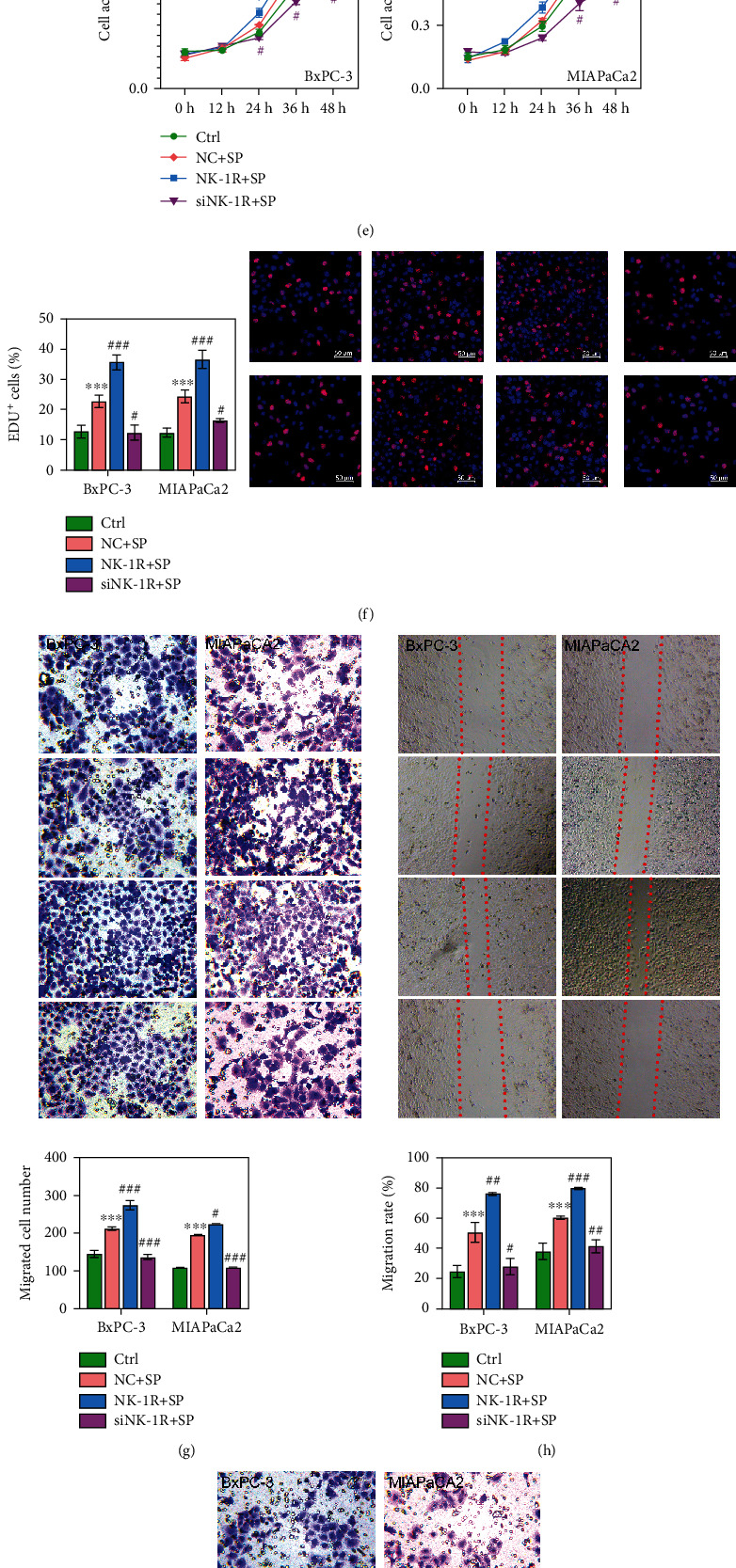
NK-1R positively regulated the proliferation, migration, and invasion of pancreatic cancer cells. (a, b) The levels of NK-1R gene and protein expression in the Ctrl, NC + SP, NK-1R + SP, and siNK-1R + SP groups. (c, d) The levels of NF-*κ*B p65, AKT, and p-AKT protein expression were assessed in different groups. (e, f) Cell proliferation rates in the Ctrl, NC + SP, NK-1R + SP, and siNK-1R + SP groups were analyzed by CCK-8 and EdU staining assays. (g, h) Transwell and wound-healing assays were performed to evaluate cell migration ability. (i) Cell invasion was assessed by the Transwell assay. ^∗^*P* < 0.05, ^∗∗∗^*P* < 0.001 vs. Ctrl. ^#^*P* < 0.05, ^###^*P* < 0.001 vs. NC + SP group.

After selecting the optimal SP concentration and treatment time, we studied how SP treatment of the NK-1R affected the pancreatic cancer cell lines. As expected, NK-1R expression was significantly increased in the NK-1R + SP group (*P* < 0.05) and decreased in the siNK-1R + SP group (*P* < 0.001) as compared with the NC-SP group (Figures 4(a) and 4(b)). The levels of NF-*κ*B p65 and p-AKT protein expression were positively correlated with the concentration of NK-1R (Figures 4(c) and 4(d)).

We next evaluated the effects of the NK-1R on cell viability and proliferation. We found that the percentages of EdU positive cells were significantly increased in the NK-1R + SP group and decreased in the siNK-1R + SP group (*P* < 0.05; Figures 4(e) and 4(f)), indicating that cell proliferation was promoted by NK-1R supplementation. The migration and invasion abilities of cells in the NK-1R + SP group were significantly increased but were decreased in the siNK-1R + SP group (*P* < 0.05; Figures 4(g)–4(i)). Taken together, these results showed that SP treatment of the NK-1R promoted the proliferation, migration, and invasion of BxPC-3 and MIAPaCa-2 cells.

### 3.5. lncRNA LOC389641 Expression and TNFRSF10A mRNA Expression Were Not Affected by SP

**Figure 5:**
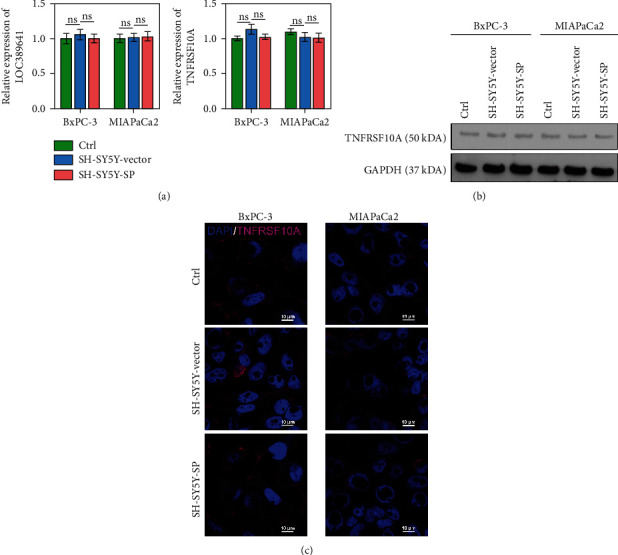
lncRNA LOC389641 expression and TNFRSF10A mRNA expression were not affected by SP. (a) The levels of lncRNA LOC389641 expression and TNFRSF10A mRNA expression were evaluated by RT-PCR. (b, c) The levels of TNFRSF10A protein expression were assessed by western blotting and IF.

*TNFRSF10A* was the target gene of LOC389641, which is believed to be one of the lncRNAs that regulates the metastasis of pancreatic cancer cells. Therefore, we evaluated the dysregulation of LOC389641 and *TNFRSF10A* in cocultured cells in the SH-SY5Y and SP groups. lncRNA LOC389641 expression was evaluated by qRT-PCR, and TNFRSF10A expression was assessed by qRT-PCR, western blotting, and IF. The levels of LOC389641 and TNFRSF10A expression showed no significant differences among groups (Ctrl, SH-SY5Y-Vector, and SH-SY5Y-SP groups) (*P* > 0.05; Figure 5(a)). As observed in Figures 5(b) and 5(c), the levels of TNFRSF10A expression also did not differ among groups.

### 3.6. Overexpression and Silencing of LOC389641 Changed the Effect of SP Stimulation on Pancreatic Cancer PNI

**Figure 6:**
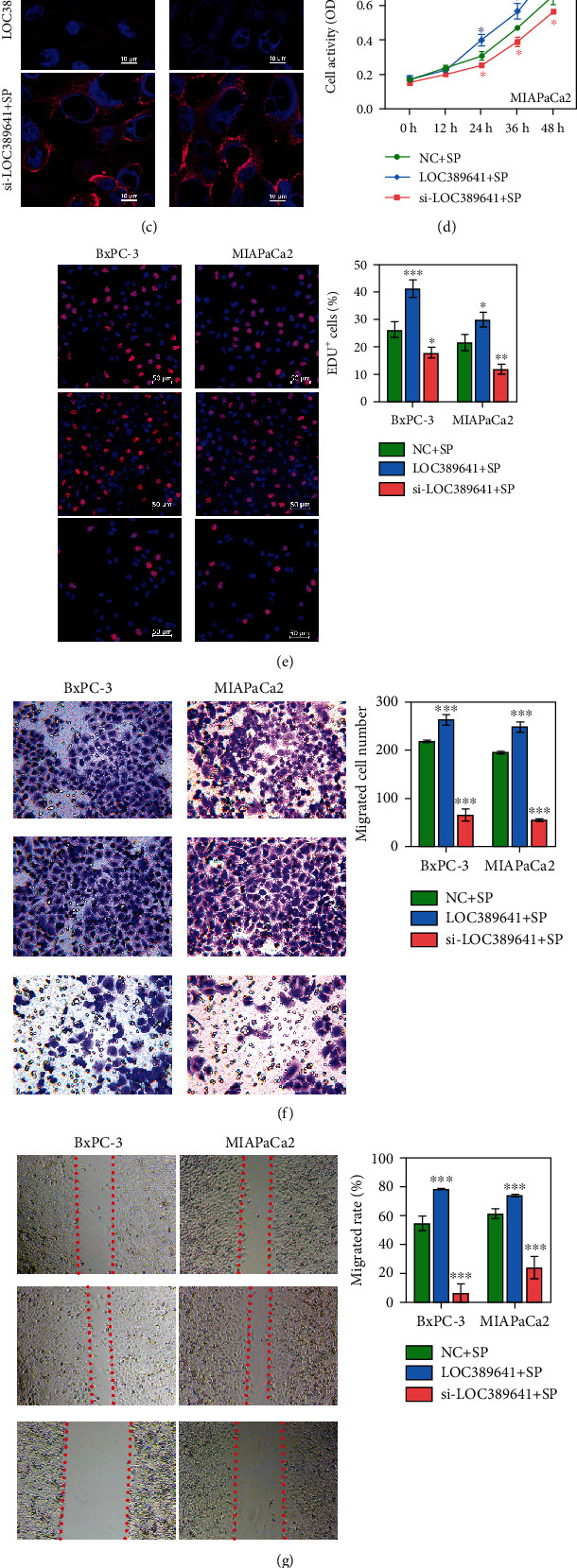
Overexpression of LOC389641 promoted the proliferation, migration, and invasion of pancreatic cancer cells. (a) The levels of LOC389641 and *TNFRSF10A* expression in the NC + SP, LOC389641 + SP, and si-LOC389641 + SP groups were evaluated by qRT-PCR. (b, c) Protein expression was assessed by western blotting and IF. (d, e) Cell proliferation rates for the NC + SP, LOC389641 + SP, and si-LOC389641 + SP groups were analyzed by CCK-8 and EdU staining assays. (f, g) Transwell and wound-healing assays were performed to evaluate cell migration ability. (h) Cell invasion was assessed by the Transwell assay. ^∗^*P* < 0.05, ^∗∗∗^*P* < 0.001 vs. NC + SP group.

We assessed the roles of LOC389641 and TNFRSF10A in the pancreatic cancer cell lines by forcing their overexpression or silencing and then culturing the cancer cells with SP. As shown in Figure 6(a), LOC389641 expression was significantly upregulated in the LOC389641 + SP group and downregulated in the si-LOC389641 + SP group (*P* < 0.001). As the target gene of LOC389641, *TNFRSF10A* showed the opposite trends in expression (Figures 6(a)–6(c)).

Next, we assessed the effects of LOC389641 overexpression or silencing on pancreatic cancer cell proliferation, migration, and invasion. Our data showed that cell viability and proliferation were significantly promoted by LOC389641 overexpression and inhibited by LOC389641 silencing (*P* < 0.05; Figures 6(d) and 6(e)). As observed in Figure 6(f), the percentage of migrated cells was significantly increased in the LOC389641 + SP group and decreased in the si-LOC389641 + SP group (*P* < 0.001). The results of wound-healing assays for cell migration were consistent with those of Transwell assays (Figure 6(g)). Furthermore, the number of invaded cells in the LOC389641 + SP group was significantly higher than in the si-LOC389641 + SP group (*P* < 0.001) (Figure 6(h)). Taken together, these data showed that either overexpression or silencing of LOC389641 could change the effect of SP stimulation on pancreatic cancer PNI, and the effect might occur by altering *TNFRSF10A* expression.

## 4. Discussion

**Figure 7:**
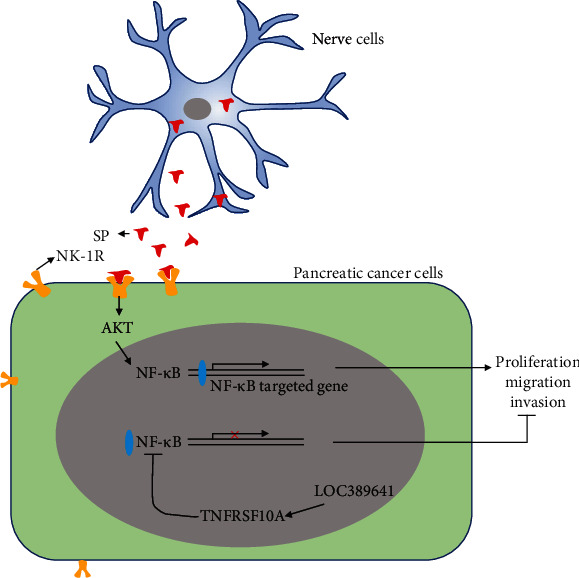
Diagram showing how the SP/NK-1R axis and lncRNA LOC389641 function in pancreatic cancer.

As the most prominent characteristic of pancreatic cancer, PNI provides an alternative route for metastatic spread and pain generation. Moreover, it is associated with the poor prognosis for patients with pancreatic cancer [17]. PNI also occurs some other cancers, including head and neck cancer [18], cervical cancer [19], rectal cancer [20], and prostate cancer [21]. Huang et al. [9] and Ayala et al. [22] used the prostate cancer/dorsal root ganglia *in vitro* system to demonstrate that PNI mechanisms involve active and reciprocal interactions between carcinoma cells and adjacent nerve/ganglia in prostate cancer. However, the extensive studies conducted on PNI and pancreatic cancer progression remain inadequate. In the present study, we further examined the roles of SP/NK-1R and lncRNA LOC389641 in pancreatic cancer PNI. Our results support the following conclusions: (1) The SP/NK-1R positively regulates the proliferation, migration, and invasion of pancreatic cancer cells. (2) Either overexpression or silencing of LOC389641 can change the effect of SP stimulation on pancreatic cancer cells by regulating TNFRSF10A expression (Figure 7). Our present study, for the first time, revealed the roles played SP/NK-1R and LOC389641/TNFRSF10A in the PNI of pancreatic cancer cells and might provide some novel biomarkers for monitoring the effect of pancreatic cancer therapy.

As one of the most common members of the tachykinin neuropeptide family, SP functions through the G protein-coupled receptors of NK-1R, -2R, and -3R [23]. SP plays roles in neurogenic inflammation, wound healing, pain transmission, and vasodilatation. By activating NK-1R, it can induce tumor-initiating intracellular responses in various cancers, including lung cancer [24], retinoblastoma [25], glioblastomas [26], breast cancer [27], and pancreatic cancer [28], and is correlated with cancer development and progression. The SP/NK-1R can substantially promote the activation of NF-*κ*B, which positively regulates transcription factors that favor tumorigenesis. Researchers have conducted numerous studies on the role of SP/NK-1R in pancreatic cancer. For example, Muñoz and Coveñas [13] demonstrated that the SP/NK-1R system is involved in the development of pancreatic cancer and might be a promising therapeutic target. Our present study found that the proliferation of pancreatic cancer cell lines cultured with human neuroblastoma cell lines was significantly regulated by SP and NK-1R. Moreover, this finding not only applies to pancreatic cancer but also to some other cancers. For example, SP/NK-1R was found to promote gallbladder cancer cell proliferation and migration [29]. Dong et al. [30] demonstrated that SP/NK-1R activation increases the growth and migration of esophageal squamous cell carcinoma cells. Moreover, the SP/NK-1R promotes the development and progression of oral cancer [31]. Although the role of the SP/NK-1R has been widely studied, its underlying mechanisms have not been fully elucidated. Some studies have found a correlation between the SP/NK-1R and chronic inflammation [32, 33]; however, that correlation has not been confirmed.

The SP/NK-1R not only promotes the progression of pancreatic cancer but also upregulates NF-*κ*B, p65, and p-AKT. A previous study found that inhibition of the NK-1R/Akt/NF-*κ*B signaling pathway could eliminate the proliferative effect of SP on gallbladder cancer [29], suggesting that NK-1R exerts a major effect on tumor growth via Akt/NF-*κ*B. The findings in that study were consistent with those in our present study, in which SP significantly promoted the NK-1R/Akt/NF-*κ*B signaling pathway. The Akt/NF-*κ*B-associated signaling pathways were promoted by mRNAs, microRNA (miRNAs), and lncRNAs in pancreatic cancers. Other studies showed that the Akt/NF-*κ*B signaling pathway was promoted by CCN1 [34] and miRNA-23b-3p [35] and involved in the tumorigenicity of pancreatic cancer. Our present study suggests that the NK-1R/Akt/NF-*κ*B signaling system affecting PNI might be a novel target for pancreatic cancer therapy.

Increasing numbers of lncRNAs have been shown to play essential roles in cancer progression. LOC389641 was reported to promote pancreatic ductal adenocarcinoma progression and increase cell invasion by regulating E-cadherin, with the possible involvement of TNFRSF10A [16]. In addition, LOC389641 has also been reported to play an important role in lung cancer [36] and thyroid cancer [37]. In the present study, we found that the coculture of LOC389641 with SP had no impact on pancreatic cancer PNI. However, either the overexpression or silencing of LOC389641 changed the effect of SP stimulation on pancreatic cancer PNI by regulating TNFRSF10A. Cytoplasmic TNFRSF10A has been considered to be a positive prognostic marker in pancreatic ductal adenocarcinoma [38], and the *TNFRSF10A* gene is involved in the NF-*κ*B signaling pathway. *TNFRSF10A* encodes for TRAILR1, which is a TNF-related apoptosis-inducing ligand receptor that can induce NF-*κ*B activation [39]. Jeong et al. [40] suggested that activated AKT regulates NF-*κ*B activation. Therefore, we assumed that pancreatic cancer PNI process promoted by the SP/NK-1R axis could be blocked by the TNFRSF10A/NF-*κ*B pathway mediated by LOC389641. Our present study does have limitations. Two such limitations are that SP and LOC389641 were not detected in clinical tissue samples, and the effects of the SP/NK-1R axis and LOC389641 on pancreatic cancer PNI lack validation in animal experiments. Additional research is needed to learn how to suppress PNI in pancreatic cancer.

## 5. Conclusion

In conclusion, our results suggest that SP stimulated the NK-1R/Akt/NF-*κ*B signaling pathway in pancreatic cancer cells cocultured with SH-SY5Y. Moreover, both NK-1R and LOC389641 individually promoted pancreatic cancer cell proliferation. We speculate that the pancreatic cancer PNI process promoted by the SP/NK-1R axis can be blocked by the TNFRSF10A/NF-*κ*B pathway mediated by LOC389641.

## Data Availability

The data used to support the findings of this study are included within the article.

## Supplementary Materials

Supplementary Materials Table S1: the primers used for qRT-PCR.
