# Supplementary material for: A Substance P (SP)/Neurokinin-1 Receptor Axis Promotes Perineural Invasion of Pancreatic Cancer and Is Affected by lncRNA LOC389641
Source: J Immunol Res. 2022 May 12;2022:5582811. doi: 10.1155/2022/5582811 (PMC9119758; doi:10.1155/2022/5582811)
Supplement: Supplementary Materials — Table S1: the primers used for qRT-PCR. [file 5582811.f1.docx]

**Table S1. The primer lists of qRT-PCR.**

| **Genes** | **Primers (5**'**-3**'**)** |
| --- | --- |
| NK-1R | Forward primer: AGGTTCCGTCTGGGCTTCAA  Reverse primer: TCCAGGCGGCTGACTTTGTA |
| LOC389641 | Forward primer: CTCAACTCCACCGTCCCTG  Reverse primer: AGAGGAACCGCTGGAAGGA |
| TNFRSF10A | Forward primer: ACCTTCAAGTTTGTCGTCGTC  Reverse primer: CCAAAGGGCTATGTTCCCATT |
| β-actin | Forward primer: CTCCATCCTGGCCTCGCTGT  Reverse primer: GCTGTCACCTTCACCGTTCC |
